# Dietary Diversity Score Associated With a Reduced Risk of Breast Cancer: A Large Population-Based Incident Case–Control Study

**DOI:** 10.1016/j.cdnut.2026.107675

**Published:** 2026-03-13

**Authors:** Ibrahim Abdollahpour, Parvane Saneei, Zahra Moradmand, Maryam Yazdi, Shaghayegh Haghjooy Javanmard, Kazem Zendehdel, James R Hebert, Torsten Bohn, Farhad Vahid

**Affiliations:** 1 Child Growth and Development Research Center, Research Institute for Primordial Prevention of Non-communicable Disease, Isfahan University of Medical Sciences, Isfahan, Iran; 2 Department of Community Nutrition, School of Nutrition and Food Science, Nutrition and Food Security Research Center, Isfahan University of Medical Sciences, Isfahan, Iran; 3 Institute for Primordial Prevention of Non-communicable Disease, Isfahan University of Medical Sciences, Isfahan, Iran; 4 Personalized Medicine Research Center, Endocrinology and Metabolism Clinical Sciences Institute, Tehran University of Medical Sciences, Tehran, Iran; 5 Cancer Biology Research Center, Cancer Institute, Tehran University of Medical Sciences, Tehran, Iran; 6 Cancer Prevention and Control Program and Department of Epidemiology and Biostatistics, Arnold School of Public Health, University of South Carolina, Columbia, SC, United States; 7 Department of Nutrition, Connecting Health Innovations LLC (CHI), Columbia, SC, United States; 8 Nutrition and Health Research Group, Department of Precision Health, Luxembourg Institute of Health, Luxembourg

**Keywords:** breast cancer, cancer risk factors, dietary diversity score, food groups

## Abstract

**Background:**

Breast cancer (BC) is responsible for a high proportion of cancer-related morbidity and mortality. A varied diet may play a role in the onset of BC.

**Objectives:**

We aimed to investigate the association between dietary diversity and BC risk.

**Methods:**

This population-based case–control study was conducted between May 2021 and October 2023, comprising 600 incident BC cases and 600 general population controls. We employed a valid and reliable 168-item food frequency questionnaire, with data collected one year prior to the date of diagnosis for cases and within the past year for controls. A dietary diversity score (DDS), focusing on consuming a variety of 5 food groups, with attainable scores between 0 and 10, was created as an indicator of total nutritional quality. Potential confounders were also assessed, including educational year, menopause, age at menarche, family socioeconomic status during adolescence, multivitamin intake, and benign breast diseases. We employed logistic regression models to estimate crude and adjusted odds ratios (ORs), controlled for potential confounders, to estimate the effect of DDS on BC risk.

**Results:**

DDS was associated with BC odds when analyzed as both a continuous and a categorical variable. The OR for DDS as a continuous variable was 0.90 [95% confidence interval (CI): 0.82, 0.99]. For participants with a DDS between 2.5 and 6, the OR was 0.58 (95% CI: 0.36, 0.95), and for those with a DDS >6, the OR was 0.41 (95% CI: 0.23, 0.73). A clear dose–response association was also observed [test for trend: OR= 0.66 (0.49, 0.86)].

**Conclusions:**

We identified DDS, a measure of a balanced diet, as a novel protective factor for BC. Given the global increase in BC morbidity and mortality, this finding underscores the need for public health interventions and educational programs targeting diverse and balanced dietary patterns.

## Introduction

Breast cancer (BC), the leading global cause of cancer mortality in females, has become the most common type of female cancer [[1], [2], [3]]. A considerable number of years of life lost [14.9 million disability-adjusted life years (DALYs)] have been attributable to BC [4,5]. Evidence also demonstrated a consistent, dramatic increase in DALYs, as well as incidence and mortality rates of BC in recent decades [1,[6], [7], [8], [9]]. For example, during the last 3 decades, the age-standardized DALYs lost, along with the incidence and mortality rates of female BC, were suggested to have increased by 19.5%, 90.9%, and 24.0%, respectively [6,10]. In line with global trends, a similar increasing pattern in the burden of BC during the same time period has also been reported in Iran [11].

In low- and middle-income countries with apparent resource limitations, it is especially important to identify risk factors that can be employed for the primary prevention of cancer. Importantly, apart from previously demonstrated risk factors, including environmental [[12], [13], [14], [15]] and genetic factors on the onset of BC, newly appearing risk and protective factors also merit further research. The role of exposure to environmental pollutants [16], including, for example, cadmium [17], perfluoroalkylated substances [18], and furthermore psychosocial stressors [19] in BC onset, for example, has been considered recently. Furthermore, dietary aspects, which could impact on inflammation [20] and oxidative stress [21], both hallmarks of cancer development, have also been related to cancer incidence.

A number of dietary indices have been demonstrated to play a role in BC development. For example, healthy dietary patterns characterized by the Portfolio Diet score [22], adherence to Mediterranean dietary patterns [23,24], and the Mean Adequacy Ratio [25] may decrease the risk of developing BC. However, processed meat [26], high-fat diets [27], and a proinflammatory diet [28] can increase the odds of BC, presumably by increasing oxidative stress and inflammation and/or altering transcription factors or nuclear receptors [29,30]. The dietary diversity score (DDS) quantifies diversity within and between food groups (5 are considered) based on a healthy, balanced diet consumed over a reference period [31]. The DDS emerged as a practical indicator of total nutritional quality within populations of different age groups and countries. Although a higher DDS has been associated with improved diet quality and a healthier diet [32,33], it also indicates that essential nutrient requirements are met [34]. Evidence suggests a potential positive role for DDS in various health outcomes [35,36], including several types of cancer [33,34]. However, the findings have not been consistent for Iranian populations, resulting in not always clear and significant findings of this indicator and cancer risk [35,36].

Increased intake of healthier food groups may significantly lower BC risk in those with a higher DDS. It is very likely, for example, that a higher DDS characterized by a higher diversity of fruits and vegetables, due to the higher intake of dietary fiber or bioactive plant metabolites [37], could reduce the odds of colorectal cancer and adenoma. Additionally, a higher diversity of dairy products may decrease the risk of colorectal cancer, whereas a higher diversity of grains may increase it [35]. Indeed, in a study on an Iranian population, a higher DDS, and a higher diversity of consumed fruit and vegetable groups were significantly related to lower odds of prostate cancer, whereas a greater diversity of meat group was directly associated with increased odds of this cancer [36], likely as meat in general, but mainly processed meats and also red meats have been related to increased risk of cancer [35,36]. However, there is little, if any, study that has evaluated the association of DDS and BC in Iranian females. Employing a large population-based incident case–control study with known sampling fractions for both case and control groups, we aimed to investigate the possible role of DDS in BC risk, while accounting for common confounding factors, including socioeconomic factors.

## Methods

This was a large case–control study of 600 incident BC cases and 600 general population controls between May 2021 and October 2023 in Isfahan, Iran [38,39]. The study base consisted of women aged 18–75 y residing in the 15 municipalities of Isfahan, the third-most populous city in Iran. A total of 1200 women aged 18–75 y were recruited from 15 municipal areas of Isfahan, including 600 incident BC cases and 600 general population controls from the same source population of cases. Data were collected using a telephone-based interview method, and verbal informed consent was obtained prior to the study participants’ inclusion in the study. Ethical approval was received by the Ethics Committee of Isfahan University of Medical Sciences (IR.MUI.REC.1399.010).

### Breast cancer case selection

The incident cases were residents of the greater Isfahan region with a confirmed diagnosis of BC who were listed in the Isfahan Breast Cancer Registry [40]. A confirmed positive pathology was available for all registered patients. We defined cases as incidents if their diagnosis occurred after the start of the study period (i.e., May 2021). All incident BC cases registered in the Isfahan Breast Cancer Registry met the inclusion criteria if: *1*) residency within the 15 municipal areas of Isfahan, and *2*) diagnosis occurring after the start of the study period. For newly diagnosed cases, the exact date of identification (year and month) was defined as the index date. During the 2.5-y study period, 653 incident cases with confirmed diagnoses of BC were registered in the Isfahan Breast Cancer Registry. Out of these, 600 (92.0%) agreed to participate and completed the study questionnaire.

### Control selection

The study population controls were women aged 18–75 y who were free of BC. They were also residents of the 15 municipality areas of Isfahan, the source population of the study. We employed a standard random-digit-dialing (RDD) protocol [41] to select the study controls from the same study base of BC cases between 2021 and 2023 (nearly 780,000 women). Accordingly, we randomly added 4 digits to the pre-codes of 15 municipalities in Isfahan. The effectiveness, utility, and feasibility [[41], [42], [43]] of the RDD approach have been well-demonstrated previously. Moreover, its comparability with address-based selected controls has also been approved [44].

### Random-digit-dialing protocol

**FIGURE 1:**
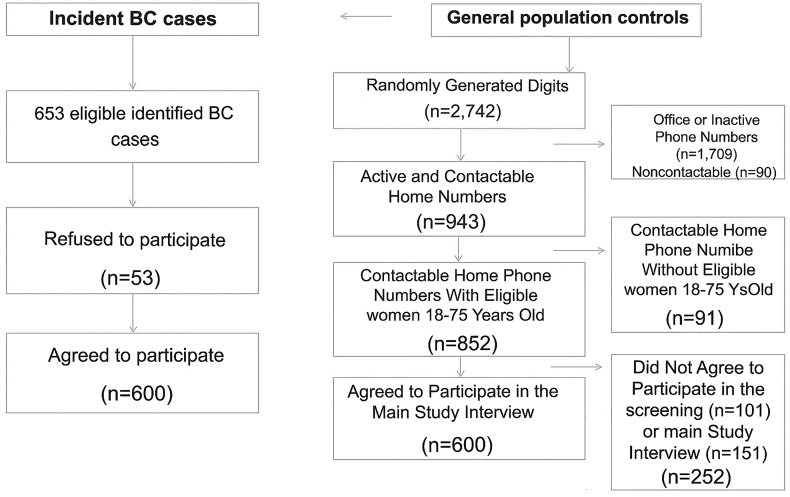
Recruitment flowchart of BC incident cases and general population female controls for the study of the association between DDS and breast cancer risk in Isfahan, Iran, 2021–2023. BC, breast cancer; DDS, dietary diversity score.

We generated 2742 random digits throughout the RDD process. According to the RDD protocol, only contactable residential numbers linked to eligible women aged 18–75 y were included. In total, 852 random residential phone numbers with eligible women were obtained (31% of the total randomly generated digits) (Figure 1). To maximize the response rate among eligible controls, we discarded a randomly generated phone number only after a predetermined number of calls (8 in total, with 2 made in the morning, 2 in the afternoon, and 4 at different times on other days) were made. Moreover, we conducted screening interviews to identify households with an eligible member before the main study interviews.

Whenever >1 eligible individual in the selected household was present, the Kish method of random sampling was used to select the study participants [45]. The Kish method is a valid and well-known process with detailed steps for randomly selecting participants from households with multiple eligible potential participants. In the current study, we exclusively recruited females. According to the Kish protocol, the higher ranks were assigned to older females. Thereafter, using the Kish method, we randomly selected one individual from each household [45]. A detailed recruitment flow chart of the study controls is shown in Figure 1. We randomly generated 2742 phone numbers during the RDD process. Of these, 1799 (65.6%) did not meet the inclusion criteria; that is, they were inactive, business/office numbers, or noncontactable. There were no eligible women aged 18–75 y in 91 of the 943 active resident households. Of the 852 remaining numbers, we were unable to convince 252 households to participate in the primary screening interview (*n* = 101) or the main study interview (*n* = 151). Finally, 600 of the randomly dialed respondents were included as the study controls and completed the study main questionnaire (response rate = 70.4%) (Figure 1).

### Data collection protocol

We developed a detailed data collection protocol to standardize the data collection process and confirm the quality of the collected data. The study protocol was initially developed to *1*) minimize the possibility of interview bias and *2*) maximize the response rate of the study's intended sample. A standardized data collection procedure was conducted, employing 5 trained female interviewers selected after a comprehensive training period for their strong interview and communication skills. At the start of the study, main interviews were conducted. To increase the response rate, the study objectives and potential benefits were explained to both the study case and control groups. To detect any potential interviewer bias, we also closely monitored the data collection process by randomly recording and reviewing interviews.

### Measurements

#### Dietary intakes and dietary diversity score

We employed a reliable and valid food frequency questionnaire (FFQ) comprising 168 food items with standard serving sizes for the Iranian population [46]. For reasons related to temporality, data on usual dietary intake were collected during the year preceding the diagnosis and 1 y prior to the interview date for the case and control groups, respectively. The FFQ used in our study was designed to capture participants’ habitual dietary intake over the previous year and thus inherently accounts for seasonal variations in the availability and consumption of foods, including carotenoid-rich fruits and vegetables. The average frequency of consumption (per day, week, or month), as well as the standard serving size of each food item, was determined using household scales, and the data were transformed into the average gram weight of daily intakes [47]. We used Nutritionist IV software (based on the USDA food database [48]) to calculate daily energy, micronutrient, and macronutrient intake for all participants.

#### Dietary diversity score calculation

We employed the method described by Kant et al. [33] for calculating dietary diversity. This was based on all 5 food groups in the USDA Food Guide Pyramid: grains, vegetables, fruits, meats, and dairy. The grains group consisted of the following 7 components: refined bread, pasta, whole-grain bread, corn flakes, biscuits, refined flour, and rice. Fruits included both fresh fruit and fruit juice, as well as berries and citrus fruits. For vegetables, we categorized them into the following groups: potatoes, other starchy vegetables, tomatoes, legumes, yellow vegetables, green vegetables, and miscellaneous vegetables. The meat group consisted of red meat, poultry, fish, and eggs. Finally, the dairy products included milk, yogurt, and cheese.

For each food group, subjects with intake above the median were scored 1; otherwise, they were scored 0. Median cut-off values were calculated separately for each food item based on daily intake (grams/day) derived from the FFQ, using the combined sample of cases and controls. Zero intake was considered below the median when applicable. Then the scores for components in each food group were summed up to create a total score for that food group. In the next step, we divided the total scores obtained in each group by the number of components in that group. Based on more recent literature [49], this value was multiplied by 2 to reflect better dietary diversity specific to our population and research context, and the total DDS for each participant was finally computed by adding the figures for the different food groups. For example, in the grains group, if a person had dietary intakes of whole-grain bread, pasta, and rice above the median, the participant’s score was calculated as (3/7) × 2 = 0.86. Therefore, the diversity score for the grains group for that person would be 0.86. After computing the diversity score for the other 4 groups, the total DDS was computed for each person. Therefore, the minimum and maximum scores for total dietary diversity among study participants could range from 0 to 10.

#### Benign breast diseases

Data on the history of benign breast diseases was obtained by asking, “Have you been diagnosed by a physician with phyllodes tumor, papilloma, fibroadenoma, cyst, fibrosis, or adenosis”? We also asked about age at diagnosis among cases who responded positively. Furthermore, data on any benign breast disease symptoms were collected to ensure validity.

#### Menopause

We confirmed the participants’ menopausal status by asking, “During the last year, are you menopausal?” For women with BC, we asked the participants to state their menopause status 1 y before BC diagnosis.

#### Family socioeconomic status during adolescence

Family socioeconomic status (SES) during adolescence was measured utilizing a 10-point visual ladder. Participants were asked to indicate their family's SES during adolescence on a 10-step visual scale depicting Isfahan's social strata. The higher the stairs, the better the education, job, and income level [50].

#### Multivitamin intake (times/week)

Information on average multivitamin intake was collected by asking: “During the past year, on average, how many times a week have you used multivitamin supplements?”

Data on educational year, age, marriage status, age at menarche, number of children, energy intake, history of BC in first-degree relatives, physical activity (MEST), BMI (in kg/m^2^), and lifetime history of any hormone use, that is, hormone replacement therapy and/or oral contraceptives, were also obtained.

### Sample size calculation

Although the mean (SD) of DDS in BC cases and controls has not been reported previously, we used the nearest study reporting the mean (6.78) and SD (1.12) of DDS among Iranian female youth as a proxy for the study controls [51]. Based on the available information, and if we assume that a 0.22 unit difference in DDS is clinically important and may decrease the risk of BC, assuming the equality of variance, at the type-1 error of (α = 5%) and with the study power of 90% (β = 10%), employing the below sample size formula for comparison of 2 mean in case–control studies, we need *n* = 545 in each study groups.

$$n=\frac{{\left(z_{1-\alpha /2}+z_{1-\beta /2}\right)}^{2}}{{\left(\mu_{1}-\mu_{2}\right)}^{2}}$$

### Statistical analysis

General characteristics of case and control groups were presented as means (SD) for continuous variables and as frequencies (percentages) for categorical variables. The study participants were classified into 3 categories of DDS score (<2.5, 2.5**–**6, >6). We employed simple and multivariable logistic regression models to estimate crude and adjusted odds ratios (ORs) and 95% confidence intervals (CIs) for DDS in relation to BC using STATA software (version 12; StataCorp, College Station, TX, USA). Several variables were considered for their potential role as confounders by examining the following criteria: ***1***) having a causal association with BC, as confirmed by current literature; ***2***) not being an intermediate factor on the causal pathway between DDS and BC; and *3*) being associated with DDS within the control group. Moreover, we checked whether the potential confounder could introduce a ∼10% change in the estimated coefficient [52]. This included age, years of education, family SES during adolescence, marital status, menopause (yes/no), age at menarche, history of hormone use (yes/no), multivitamin intake (time/week), and history of benign breast diseases (yes/no). Replacing the categorical DDS with a single predictor and using category rank scores, we performed a test for the trend of DDS as an ordinal categorical variable. Finally, the whole analysis was performed stratified by menopausal status.

## Results

As demonstrated in Table 1, cases were more highly educated than controls (12.4 vs. 9.5, *P* < 0.001). The subjects’ family SES during adolescence in the cases was significantly higher than in the controls (5.3 vs. 4.5, *P* < 0.001). Although 35.2% of controls were menopausal, 54.5% of cases had a similar experience (*P* < 0.001). Moreover, the mean multivitamin intake in controls was significantly higher than in cases (0.31 vs. 0.15, *P* < 0.001).

**TABLE 1:** Characteristics of breast cancer cases and general population controls, Isfahan, 2021–2023

	Cases (*n*%)	Controls (*n*%)	Crude OR, 95% CI	*P* value
Age (y); mean (SD)	50.0 (9.7)	45.9 (12.9)	1.03 (1.02, 1.04)	0.001
Age				
< 40	101 (16.83)	216 (36.12)	1	-
40–50	224 (37.33)	158 (26.42)	3.03 (2.22, 4.14)	<0.001
> 50	275 (45.83)	224 (37.46)	2.62 (1.96, 3.53)	<0.001
Marriage status				
Married	480 (80.13%)	470 (78.46%)	1.11 (0.84, 1.46)	0.48
Educational years	12.39 (4.47%)	9.52 (5.05%)	1.13 (1.10, 1.16)	<0.001
Menopause				
Yes	327 (54.40%)	211 (35.17%)	2.21 (1.75, 2.78)	<0.001
Age at menarche	13.23 (1.73)	13.55 (1.67)	0.89 (0.84, 0.96)	0.001
History of hormone use				
Yes	285 (47.58%)	265 (44.17%)	1.15 (0.91, 1.44)	0.24
Family SES during adolescence	5.31 (1.87)	4.5 (2.04)	1.22 (1.14, 1.29)	<0.001
BMI (kg/m^2^); mean (SD)	27.10 (4.81)	26.76 (4.63)	1.01 (0.99, 1.04),	0.22
Multivitamin intake (Times/week)	0.15 (0.55)	0.31 (0.80)	0.68 (0.55, 0.83)	<0.001
History of benign breast diseases				
Yes	170 (28.62%)	123 (20.74%)	1.53 (1.17, 2.00)	0.002

CI, confidence interval; OR, odds ratio; SD, standard deviation; SES, socioeconomic status.

We further attempted to examine the relation of individual food groups and the risk of BC. Table 2 displays the results of logistic regression models examining both unadjusted and adjusted associations between the diversity of the 5 main dietary groups included in the DDS and BC risk. Notably, among these diet groups, meat diversity consistently showed a significant inverse association with BC odds after adjustment for the study’s main confounders. In the multivariable logistic model, those with a meat diversity score above the median had 42% lower odds of BC than those with a score below the median (OR: 0.58; 95% CI: 0.42, 0.80). A similar finding was observed when treating the meat diversity score as a continuous variable (OR: 0.64; 95% CI: 0.49, 0.84) and when considering meat intake per weight (OR: 0.99; 95% CI: 0.993, 0.999). No statistically significant associations were found between other food group diversity scores and BC development (Table 2).

**TABLE 2:** Unadjusted and adjusted associations between the 5 main groups of the dietary diversity score and breast cancer, based on the study in Isfahan, 2021–2023

	Cases (*n* = 600)	Controls (*n* = 600)	Unadjusted OR, 95% CI, *P* value	Adjusted OR, 95% CI, *P* value^1^
Meat (diversity); mean (SD)	0.97 (0.62)	1.13 (0.62)	0.66 (0.55, 0.79), <0.001	0.64 (0.49, 0.84), 0.001
Meat (diversity); median				
<1	206 (34)	279 (47)	1	1
≥1	392 (66)	321 (54)	0.60 (0.48, 0.76), <0.001	0.58 (0.42, 0.80), 0.001
Meat (g); mean (SD)	80.72 (60)	91.00 (58)	0.99 (0.995, 0.999), 0.003	0.99 (0.993, 0.999), 0.03
Dairy (diversity); mean (SD)	0.99 (0.74)	1.04 (0.68)	0.89 (0.76, 1.05), 0.17	0.86 (0.69, 1.08), 0.21
Dairy (diversity); median				
<1.33	309 (52)	317 (53)	1	1
≥1.33	291 (49)	283 (47)	0.95 (0.75, 1.19), 0.64	1.01 (0.74, 1.39), 0.93
Dairy (g); mean (SD)	331.32 (453)	295.94 (336)	1.00 (0.99, 1.00), 0.13	1.00 (1.00, 1.001), 0.02
Grain (diversity); mean (SD)	1.00 (0.51)	1.01 (0.46)	0.95 (0.75, 1.20), 0.67	1.06 (0.74, 1.52), 0.73
Grain (diversity); median				
<1.14	295 (49)	294 (49)	1	1
≥1.14	305 (51)	306 (51)	0.95 (0.75, 1.19), 0.64	1.12 (0.81, 1.54), 0.49
Grain (g); mean (SD)	267.24 (200)	272.65 (216)	0.99 (0.999, 1.00), 0.65	1.00 (0.999, 1.001), 0.25
Fruits (diversity); mean (SD)	0.97 (0.93)	1.03 (0.92)	0.94 (0.83, 1.06), 0.33	0.85 (0.71, 1.02), 0.08
Fruits (diversity); median				
<1	345 (58)	339 (57)	1	1
≥1	255 (43)	260 (43)	1.03 (0.82, 1.30), 0.75	0.77 (0.55, 1.07), 0.12
Fruits (kg); mean (SD)	0.42 (0.39)	0.44 (0.39)	0.86 (0.65, 1.15), 0.33	0.66 (0.39, 1.11), 0.11
Vegetables (diversity); mean (SD)	0.98 (0.60)	1.01 (0.55)	0.92 (0.97, 1.05), 0.39	0.97 (0.78, 1.21), 0.81
Vegetables (diversity); median				
<1.14	309 (52)	287 (48)	1	1
≥1.14	289 (48)	312 (52)	1.16 (0.93, 1.46), 0.19	0.99 (0.72, 1.37), 0.97
Vegetables (kg); mean (SD)	0.31 (0.28)	0.35 (0.26)	0.55 (0.34, 0.91), 0.02	0.63 (0.32, 1.26), 0.19

CI, confidence interval; OR, odds ratio; SD, standard deviation.

1 Adjusted for educational year, menopause, age, marital status, age at menarche, history of hormone use, family socioeconomic status during adolescence, age at last delivery, BMI, child number, vitamin intake, history of breast cancer in first-degree relatives, physical activity, multivitamin intake, abortion history, energy intake, and benign breast diseases.

Table 3 presents the results of logistic regression models of the underlying association between DDS and BC incidence, both unadjusted and adjusted for 2 well-known confounders. Notably, the proportion of individuals with higher DDS values (>6) in the control group was significantly higher than in the case group (*P* = 0.005). As shown, consistent significant associations were detected between DDS and BC odds after adjustment for the 2 sets of the study’s main confounders. In the full model, the odds of BC was 59% lower among those with DDS scores > 6 than among those with DDS scores 0–2.5 (OR: 0.41; 95% CI: 0.23, 0.73). A similar finding was observed for the second category, i.e., those with DDS scores between 2.5 and 6 (OR: 0.58; 95% CI: 0.36, 0.95). Notably, a clear dose–response association was observed for the DDS categories (OR: 0.66; 95% CI: 0.49, 0.86) (Table 3).

**TABLE 3:** Unadjusted and adjusted associations between dietary diversity score and breast cancer, Isfahan, 2021–2023

	Cases (*n* = 600)	Controls (*n* = 600)	OR, 95% CI	*P* value
Dietary diversity score; mean (SD)	4.92 (2.40)	5.22 (2.23)	0.94 (0.90, 0.99)^1^	0.03
Dietary diversity score (categorical)				
≤2.5	112 (18.79)	75 (12.54)	1	-
2.5–6	269 (45.13)	286 (47.83)	0.63 (0.45, 0.88)^1^	0.007
>6	215 (36.07)	237 (39.63)	0.61 (0.43, 0.86)^1^	0.005
Test for trend			0.82 (0.69, 0.96)^1^	0.015
Dietary diversity score; mean (SD)			0.95 (0.90, 1.00)^2^	0.06
Dietary diversity score (categorical)				
≤2.5			1	
2.5–6			0.64 (0.45, 0.92)^2^	0.02
>6			0.61 (0.42, 0.88)^2^	0.009
Test for trend			0.82 (0.68, 0.97)^2^	0.02
Dietary diversity score; mean (SD)			0.90 (0.82, 0.99)^3^	0.036
Dietary diversity score (categorical)				
≤2.5			1	
2.5–6			0.58 (0.36, 0.95)^3^	0.03
>6			0.41 (0.23, 0.73)^3^	0.003
Test for trend			0.65 (0.49, 0.86)^3^	0.003

CI, confidence interval; OR, odds ratio; SD, standard deviation; SES, socioeconomic status.

1 Unadjusted OR.

2 Adjusted for educational year, menopause, age, marital status, and age at menarche.

3 Further adjusted history of hormone use, family SES during adolescence, age at last delivery, BMI, child number, Vitamin intake, history of breast cancer in first-degree relatives, physical activity, multivitamin intake, abortion history, energy intake, and benign breast diseases.

Our stratified analysis by menopausal status revealed statistically significant associations between DDS and breast cancer risk in premenopausal groups; the odds of BC in those with DDS values >6 were 75% lower than in those with DDS scores of 0–2.5 (OR: 0.24; 95% CI: 0.10, 0.57). A similar finding was observed for the second category, that is, those with DDS scores between 2.5 and 6 (OR: 0.41; 95% CI: 0.24, 0.85). However, the findings for the postmenopausal group were not significant (data not shown).

## Discussion

In this large population-based incident case–control study, we found that higher DDS scores were a significant protective factor against the odds of BC. To our knowledge, this is one of the first studies to examine the association between DDS and BC. We also detected a clear dose–response association, with the magnitude of effect increasing with higher DDS: a one-unit increase in DDS decreased the risk of BC by 10%. This increase in validity supports the demonstrated association.

Our findings are consistent with those of a hospital-based case–control study conducted in Tehran, Iran, involving 149 BC cases and an equal number of controls, which reported a significant inverse association between the diversity scores of certain food groups and BC risk [53]. When comparing our results with other hormone-dependent cancers, similar protective associations have been reported. For instance, greater dietary diversity and higher-quality diets have been inversely related to prostate cancer risk in Iranian populations [36], as well as to colorectal and bladder cancer risk in other studies [35,37]. Evidence on the potential role of DDS in reducing the risk of various cancers remains limited, yet emerging data from different cancer sites support its importance. For example, Lim et al. [54] reported that gastric cancer patients in Korea had significantly poorer dietary quality compared to healthy controls, highlighting that inadequate diversity and nutrient intake may contribute to cancer risk. Similarly, a systematic review and meta-analysis by Milajerdi et al. [55] showed that higher dietary quality, as assessed by various indices, was associated with lower overall cancer mortality.

Consistent inverse associations across cancer sites suggest that dietary diversity may have protective effects beyond BC, particularly in hormone-dependent cancers. This may be partly explained by the higher intake of fiber, vitamins, minerals, and phytochemicals in more diverse diets, which can reduce oxidative stress and chronic inflammation, key processes in carcinogenesis. We note that anti-inflammatory mechanisms are proposed only as a plausible pathway and do not equate DDS with indices such as the dietary inflammatory index (DII) [56]. Additionally, the relatively low mean DDS in our population (≈5), compared with Western cohorts (often averaging 6–7), indicates that modest improvements in dietary diversity may have meaningful public health implications.

The diversity across specific food groups within the DDS likely contributes differentially to cancer risk. Notably, a higher intake of healthier food groups such as fruits and vegetables or grains might be responsible for a significant decrease in the odds of BC in those with higher DDS. As part of the composition of a variety of food items, diet diversity involves consuming different macronutrients and micronutrients, as well as secondary plant metabolites, including vitamin C and E, flavonoids, phytosterols, and carotenoids. Many of these are known to act as potent antioxidants, anti-inflammatory agents, and anticarcinogenic agents [37]. Furthermore, a more diverse diet may be linked to a richer diet and a wider range of micro- and macronutrients, ensuring the presence of all essential nutrients, which has also been recognized as a protective factor against cancer [57]. A critical component is dietary fiber, which has been linked to anti-inflammatory effects, for example, by promoting the production of short-chain fatty acids [58]. Also, fiber intake has been associated with impaired estrogen metabolism, increased systemic inflammation, and altered gut microbiota, all mechanisms linked to breast carcinogenesis [59].

Conversely, red and processed meats, while providing essential nutrients such as iron, may increase cancer risk by generating reactive oxygen species via the Haber-Weiss reaction, thereby promoting oxidative stress and DNA damage [60]. Interestingly, and consistent with this, among the main dietary diversity components assessed, only the diversity of the meat group showed a statistically significant association with BC risk, whereas the diversity scores of fruits, vegetables, dairy, and grains did not show independent protective effects. Meat can be consumed in a wide variety of items, including red meat and poultry, as well as processed meats such as sausages. It is thought that some types of meat may more likely foster certain types of cancer, for example, red meat due to their higher heme iron content and nitrites from processed meats and risk of BC was reported earlier [61], whereas fish and poultry were, in most studies, not found to be related to increased BC risk in earlier studies [[62], [63], [64]], possibly due to their lower iron content and more healthy fatty acid composition. In this respect, greater diversity may reflect a more prudent and balanced approach to consuming various sources of these different meat groups. However, meat diversity should be interpreted cautiously, as prior evidence suggests differential effects of meat subtypes on BC risk [63,[65], [66], [67]].

The inverse association observed for meat diversity should be interpreted with considerable caution. The DDS meat component aggregates heterogeneous subtypes, including red meat, processed meat, poultry, fish, and eggs, which have demonstrated differential associations with BC risk in previous literature. It is therefore unlikely that “meat diversity” per se is protective. Rather, the observed association may reflect a more favorable distribution of meat subtypes, such as higher consumption of fish and poultry relative to red and processed meats. Because the DDS does not distinguish between health-promoting and potentially harmful subtypes within a food group, the protective signal may represent dilution of adverse effects from red and processed meat by healthier alternatives. Accordingly, our findings should not be interpreted as evidence supporting increased consumption of red or processed meat, but rather as reflecting overall dietary balance within the broader dietary pattern. This discrepancy highlights the importance and potential advantage of using a composite dietary index, such as the DDS, rather than examining individual food groups in isolation. Although single-food-group diversity scores may capture specific aspects of dietary habits, they may not accurately reflect the overall dietary pattern and nutrient synergy required to meaningfully influence disease risk [66].

In contrast, the DDS integrates the collective contributions of multiple food groups, capturing broader dietary balance and variety that may be more indicative of long-term nutritional adequacy and protective effects. The consistent and significant inverse association between DDS and BC odds, observed across continuous and categorical models and demonstrating a clear dose–response trend, supports the utility of this comprehensive index as a more robust and informative measure. These findings suggest that relying solely on individual food group diversity may underestimate the impact of dietary patterns on disease outcomes. In contrast, composite indices, such as the DDS, offer a more holistic, policy-relevant tool for assessing and promoting dietary quality in cancer prevention efforts.

However, also the variety within a food group is also expected to benefit cancer prevention. For instance, greater variety within fruit and vegetable groups can enhance intake of antioxidants, such as polyphenols, carotenoids, and vitamins C and E, which are known to modulate transcription factors and exert anti-inflammatory, antiproliferative, and anticarcinogenic effects [68]. Many of these constituents may act synergistically, such as vitamin E and vitamin C, and adequate intake may be better ensured by varied consumption across food groups. Furthermore, low diversity in these plant-based groups may reflect insufficient fiber intake or higher sugar intake from fruits, both of which have been linked in some studies to cancer risk [[69], [70], [71]]. Similarly, a low intake of fish within the meat and fish group may be associated with limited intake of polyunsaturated and omega-3 fatty acids, which may contribute to a proinflammatory dietary milieu that exacerbates adiposity-related hormone dysregulation and cancer risk [72].

Compared with nutrient-based indices such as the DII and dietary antioxidant index (DAI) [67,72], which estimate the inflammatory or antioxidant potential of diets, the DDS uniquely reflects the variety and balance of food group intake, regardless of quantity [33]. Although DII and DAI are mechanistically oriented, DDS captures broader dietary habits and food access, making it a practical, policy-relevant tool for population-level assessment [73,74]. It is also less dependent on detailed nutrient databases and easier to apply in large-scale or resource-limited settings, including less detailed FFQs. Prior studies suggest that food-group–based indicators may reduce complexity and variance, potentially yielding stronger associations with disease outcomes [56]. The protective association observed here further supports DDS as an independent indicator of dietary quality relevant to BC risk.

Employing a random sampling method to recruit general population controls and achieving an excellent response rate among BC incident cases and a large recruited sample size, were among the study's strengths [33,56,68,[73], [74], [75]]. However, some limitations should be considered when interpreting the current study's findings. As an inherent limitation of case–control studies, recall bias in dietary intake information should not be ignored. A cancer diagnosis may subconsciously influence patients’ recall of their prior dietary habits compared with those of healthy controls. However, recruiting incident cases might alleviate the degree of important recall bias. The direction and magnitude of imposed bias are considerably influenced by the differential or nondifferential nature of misclassification. Concern about differential misclassification when estimating ORs in case–control studies is more common.

In contrast, the presence of nondifferential misclassification when detecting significant ORs would be desirable. Nevertheless, it is not straightforward to determine the direction of misclassification for the study's main exposure, i.e., DDS, and its confounders. This might be influenced by the independent or dependent nature of measurement error in the study's main exposure and confounders [52]. The probability of selection bias is another limitation of case–control studies that should be noted. Furthermore, random-digit dialing can capture only those subgroups of the study base with residential phone lines [41]. As essential requirements for active, contactable residential lines, a steady residence and a minimum household income are necessary. This may not be the case for all residents of the 15 municipalities in Isfahan, resulting in some degree of selection bias. Despite these methodological issues, excellent response rates in the case (92%) and control (70.4%) groups, along with the random nature of recruitment in the general population controls, mitigated the potential for major selection bias. However, the findings of the current study should be interpreted in light of certain limitations related to generalizability. Because the study population consisted of women residing in urban areas of Iran, the results may not be directly applicable to rural populations or to women in other countries with different dietary patterns, socioeconomic conditions, and cultural contexts. Dietary diversity and its determinants vary substantially across regions, influenced by food availability, traditional cuisines, and lifestyle factors. Therefore, the observed association between DDS and BC risk may differ in settings with distinct nutritional profiles or health systems. Moreover, although the DDS effectively captures dietary diversity across food groups, it primarily reflects variety rather than the nutritional composition or quality of foods within each group. Importantly, DDS does not differentiate between healthy and less healthy food choices within the same category (e.g., whole grains vs. refined grains), which may influence the interpretation of its association with BC risk. This limitation is particularly relevant for the meat group, where aggregation of red, processed, and white meats may obscure subtype-specific associations. As another limitation, broad food-group aggregation may obscure differences in health effects among individual items, such as combining protective foods with potentially harmful ones, thereby diluting observed associations. Primarily, DDS is a qualitative measure, and the dose–response analysis using gram intake may provide additional context on the potential quantitative contribution of food groups to BC risk. This analysis is exploratory and intended to complement, rather than replace, the primary qualitative DDS analysis. Finally, although Baigi et al. [76] concluded that subjective social status, as measured by the 10-step visual ladder, can serve as a valuable indicator of SES, its estimates should be interpreted with caution.

In conclusion, considering residual confounding and recall bias as inherent limitations of case–control studies, we found that women with lower dietary diversity were at greater risk of BC development than those with higher DDS scores. This significant association, with a clear dose–response trend, remained after controlling for potential confounding variables. Based on the current study's findings, a more diverse overall dietary pattern was associated with lower BC risk. However, the observed association for meat diversity should be interpreted cautiously, given the heterogeneous effects of different meat subtypes reported in prior literature. Given nutrition's global role in population health, educational programs promoting dietary diversity are recommended for BC prevention. Our findings contribute to the epidemiologic evidence supporting dietary guidelines for a more diverse diet to protect women against BC. Furthermore, cancer prevention strategies and public health policies promoting adherence to a more diverse dietary pattern in women may help reduce BC incidence. Conducting additional longitudinal research is necessary to explore further the relationship between DDS and BC risk, as well as other health outcomes.

## Declaration of Generative AI and AI-assisted technologies in the writing process

The author(s) declare that no generative AI or AI-assisted technologies were used in the writing of this manuscript.

## Author contributions

The authors’ responsibilities were as follows – I.A. contributed to idea generation, designing study, data collection, analysis, writing, and editing the manuscript. P.S. and Z.M. contributed to idea generation, analysis, and finalizing the manuscript. M.Y. incorporated data analysis, writing, and editing the manuscript. S.H.J. assisted with data collection and manuscript finalization. K.Z. contributed to data analysis, writing, and editing the manuscript. J.R.H. contributed to idea generation, data analysis, and manuscript writing and editing. F.V. and T.B. contributed to the writing and editing of the manuscript and were responsible for the submission of the article and all authors: read and approved the final manuscript.

## Data availability

The data will be made available upon request. As the first author, Dr. Ibrahim Abdollahpour had full access to all data in the study and takes responsibility for the integrity and accuracy of the data analysis. Dr. Ibrahim Abdollahpour conducted the data analysis and is responsible for it.

## Funding

This work was supported by Isfahan University of Medical Sciences (grant number: 199061) and the Shams Charity Organization, as well as the Cancer Institute of Iran, Tehran University of Medical Sciences (grant number: 1400-2-244-53323).

## Conflict of interest

The authors declare no conflicts of interest.
